# *Rickettsia slovaca* Infection, France

**DOI:** 10.3201/eid1203.050911

**Published:** 2006-03

**Authors:** Frédérique Gouriet, Jean-Marc Rolain, Didier Raoult

**Affiliations:** * Université de la Méditerranée, Marseille, France

**Keywords:** *Rickettsia slovaca*, *Rickettsia*, tick, rickettsiosis, tick bite, Tibola, letter

**To the Editor:** *Rickettsia slovaca* was first isolated in 1968 in a *Dermacentor marginatus* tick collected in Slovakia, and serologic evidence of infection with this bacteria was reported in patients with enlarged lymph nodes and a scalp eschar after being bitten by a tick (*1*). However, the first proven case of *R*. *slovaca* infection was reported only in 1997 in France (*2*). This rickettsiosis is called tickborne lymphadenopathy (TIBOLA) because the most pronounced sign is lymph node enlargement. In Spain the same condition is called *Dermacentor*-borne-necrosis-erythema lymphadenopathy (*3**,**4*).

In this study, we describe 14 new patients with TIBOLA from southern France who sought treatment from January 2004 to May 2005 and compare the features of these patients with those in whom Mediterranean spotted fever (MSF) was diagnosed during the same period. All the patients were referred to our center with a suspected rickettsial infection characterized by a tick bite located on the scalp, an inoculation eschar, and enlarged lymph nodes (Figure A1). For each patient, an acute-phase and a convalescent-phase serum sample were obtained for serologic analysis. Culture and polymerase chain reaction (PCR) were performed on tick, skin biopsy, or blood specimens. A multiple-antigen immunofluorescence assay (IFA) was performed by using 5 spotted fever group (SFG) rickettsial antigens: *R*. *conorii conorii*, *R*. *slovaca*, *R*. *helvetica*, *R*. *sibirica mongolitimonae*, and *R*. *felis*. Titers of at least 64 for immunoglobulin G (IgG) and 32 for IgM in acute-phase serum samples, evidence of seroconversion with 4-fold increases in IgG titers, or both, were considered as evidence of recent infections with a *Rickettsia* sp. (5). For serum specimens confirmed by IFA at the species level, Western immunoblotting and cross-adsorption assays procedures were performed as described elsewhere (6) by using *R*. *conorii conorii* and *R*. *slovaca* antigens. Patients with a definite serologic diagnosis at the species level were analyzed for their epidemiologic and clinical information.

Culture from skin biopsy specimen and ticks were injected into human embryonic lung cells and cultivated into shell-vial culture as previously described (7). DNA was extracted from skin biopsy specimens, acute-phase serum samples, and ticks by using the QIAamp DNA Mini Kit (Qiagen, Hilden, Germany) (8). Standard PCR was performed with primers suitable for hybridization within the conserved region of genes coding for outer membrane protein A (*ompA*) and citrate synthase (*gltA*) (8).

Among the 14 patients in a scalp lesion and cervical or occipital (1 case) lymph node enlargement developed after they were bitten by a tick, 9 were female (1 was pregnant) and 5 were male. The median (range) age was 34.9 (5-85) years with half of the patients <10 years of age. The incubation period ranged from 5 to 15 days (median 10.5 days; n = 7). Only 3 patients had fever. All patients fully recovered with doxycycline or, for the pregnant patient, josamycin therapy. Serology confirmed the diagnosis of *R*. *slovaca* infection for 10 patients by microimmunofluorescence and Western blot analysis after cross-adsorption studies (Table A1). *R*. *slovaca* was amplified by PCR for 7 cases, including 3 skin biopsy specimens, 3 *Dermacentor marginatus* ticks, and 1 acute-phase serum sample (Table A1). Three isolates (2 from skin biopsy specimens and 1 from a tick) were obtained by using the shell-vial culture assay. During the period of our study, in the same French region, 40 patients with MSF were clinically and laboratory diagnosed using the same procedures. The median (range) age was 54.2 (5–85) years with only 3 children <10 years of age (compared to 7/7 children with *R*. *slovaca* infection, p = 0.0015). MSF occurred mainly during the summer, whereas *R*. *slovaca* infection was seen during the colder months with 6 cases from October to January and 8 cases from February to May (Figure).

In France, *R*. *conorii* has long been considered to be the only SFG rickettsiosis but *R*. *slovaca* may also be prevalent (*9*), contributing 25% of the cases in the present study. This organism is also a common cause of disease in Hungary and in La Rioja, Spain (*3*). These data suggest that TIBOLA mainly occurs in young children, affects women predominately, and occurs primarily during the colder months (*9**,**10*). As previously reported (*9*), we found that standard microimmunofluorescence serologic testing was insensitive and that Western blot is more useful and allows identification to the species level after cross-adsorption studies. Finally, DNA amplification by PCR from skin biopsy tissue, serum samples, or in ticks allowed confirmation of the diagnosis in only 50% of the cases, which suggests that other rickettsial species may be responsible for TIBOLA. Epidemiologic and clinical presentations are so characteristic that the clinical diagnosis should be considered in patients who have been bitten on the scalp during the colder months. In Europe, *R*. *slovaca* infection is likely to be a significant cause of cervical lymph node enlargement, and microbiologic investigation and tick analysis will underline the relative importance of this disease.

## Figures and Tables

**Figure:**
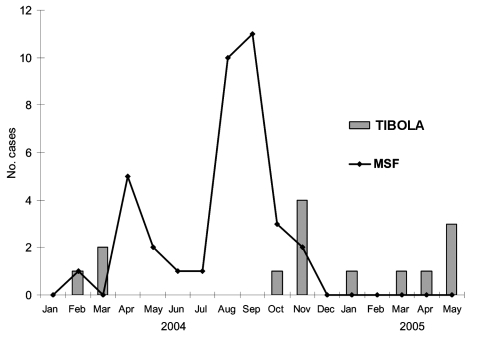
Seasonal distribution of Mediterranean spotted fever (MSF) and tickborne lymphadenopathy (TIBOLA) in southern France from January 2004 to May 2005.

**Figure A1:**
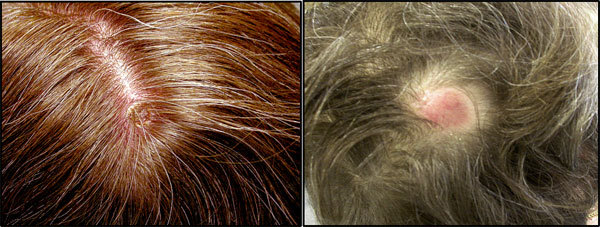
Patients with *Rickettsia slovaca* infection. On the left, inoculation lesion on the scalp; on the right, residual alpecia.

## Appendix

**Table A1:** Laboratory results for 14 patients with *Rickettsia slovaca* infection*

Patient	MIF assay				Culture		PCR		
	Acute-phase serum†	Convalescent-phase serum*	WB	Cross-adsorption	Skin biopsy	Tick	Skin biopsy	Tick	Acute phase serum
1	256/0	256/0	+	*R*. *slovaca*	–	NA	–	NA	–
2	0/32	NA	+	*R*. *slovaca*	–	NA	*R*. *slovaca*	NA	–
3	0/0	0/0	+	*R*. *slovaca*	*R*. *slovaca*	NA	*R*. *slovaca*	NA	–
4	0/0	0/0	NA	*NA*	*R*. *slovaca*	NA	*R*. *slovaca*	NA	–
5	0/0	32/32	+	*R*. *slovaca*	NA	NA	NA	NA	*R*. *slovaca*
6	0/32	0/32	+	*R*. *slovaca*	NA	NA	NA	NA	–
7	0/0	64/64	NA	NA	NA	*R*. *slovaca*	NA	*R*. *slovaca*	–
8	0/0	NA	+	*R*. *slovaca*	–	NA	–	NA	–
9	32/32	NA	+	*R*. *slovaca*	–	NA	–	NA	–
10	0/0	NA	NA	NA	NA	NA	NA	*R*. *slovaca*	–
11	0/0	NA	NA	NA	NA	NA	NA	NA	–
12	64/0	64/0	+	*R*. *slovaca*	–	–	–	*R*. *slovaca*	–
13	0/128	NA	+	*R*. *slovaca*	NA	NA	NA	NA	–
14	256/128	NA	+	*R*. *slovaca*	NA	NA	NA	NA	–

*MIF, microimmunofluorescence; WB, Western blot; PCR, polymerase chain reaction; NA, not available. †Antibody titers are immunoglobulin G (IgG)/IgM against *R*. *slovaca*.
